# Myelodysplastic syndromes are multiclonal diseases derived from hematopoietic stem and progenitor cells

**DOI:** 10.1186/s40164-022-00280-3

**Published:** 2022-05-16

**Authors:** Bingqing Luo, Fang Dong, Tiejun Qin, Qingyun Zhang, Haitao Bai, Jinhong Wang, Yujiao Jia, Shihui Ma, Erlie Jiang, Tao Cheng, Zhijian Xiao, Hideo Ema

**Affiliations:** 1 State Key Laboratory of Experimental Hematology, National Clinical Research Center for Blood Diseases, Haihe Laboratory of Cell Ecosystem, Institute of Hematology & Blood Diseases Hospital, Chinese Academy of Medical Sciences & Peking Union Medical College, Tianjin, 300020 China; 2 MDS and MPN Center, Institute of Hematology & Blood Diseases Hospital, Chinese Academy of Medical Sciences & Peking Union Medical College, Tianjin, 300020 China

**Keywords:** Myelodysplastic syndromes (MDS), Clonal hematopoiesis (CH), Cell of origin, Hematopoietic stem cells (HSCs), Hematopoietic progenitor cells (HPCs), Single-cell analysis, Targeted single-cell RNA sequencing

## Abstract

Myelodysplastic syndromes (MDS) are generally considered as a group of clonal diseases derived from hematopoietic stem cells, but a number of studies have suggested that they are derived from myeloid progenitor cells. We aimed to identify the cell of origin in MDS by single-cell analyses. Targeted single-cell RNA sequencing, covering six frequently mutated genes (*U2AF1*, *SF3B1*, *TET2*, *ASXL1*, *TP53*, and *DNMT3A*) in MDS, was developed and performed on individual cells isolated from the CD34^+^ and six lineage populations in the bone marrow of healthy donors (HDs) and patients with MDS. The detected mutations were used as clonal markers to define clones. By dissecting the distribution of clones in six lineages, the clonal origin was determined. We identified three mutations both in HDs and patients with MDS, termed clonal hematopoiesis (CH) mutations. We also identified fifteen mutations only detected in patients with MDS, termed MDS mutations. Clonal analysis showed that CH clones marked by CH mutations and MDS clones marked by MDS mutations were derived from hematopoietic stem cells as well as various hematopoietic progenitor cells. Most patients with MDS showed the chimeric state with CH clones and MDS clones. Clone size analysis suggested that CH mutations may not contribute to clonal expansion of MDS. In conclusion, MDS comprise multiple clones derived from hematopoietic stem and progenitor cells.

**Supplementary Information:**

The online version contains supplementary material available at 10.1186/s40164-022-00280-3.

To the editor,

Myelodysplastic syndromes (MDS) were proposed to arise from a stepwise acquisition of mutations [1, 2] and are reported as hematopoietic stem cells (HSCs)-origin diseases [3]. However, an increasing number of studies have suggested myeloid progenitor cells are the cell of origin [4, 5]. Additional mutations can be acquired and confer the self-renewal ability to hematopoietic progenitor cells (HPCs) in the progression of MDS [3, 4]. Thus, the cell of origin in MDS remains controversial. In principle, mutant clones that originate from HSCs should be detected in all their descendent blood lineages, enabling the reconstruction of clonal structures in cancer [6]. Single-cell sequencing but not bulk sequencing detects the co-occurrence of mutations in the same cells, permitting to distinguish individual clones from one another. In this study, we took advantage of lineage tracing techniques by using targeted single-cell RNA sequencing to address this issue [7].

BM cells were obtained from five healthy donors (HDs) and two AML patients in complete remission (HD group), as well as fourteen MDS patients (MDS group) (Additional file 1: Table S1). Single cells were sorted from CD34^+^ HPCs and six lineage populations: neutrophils, monocytes, erythroblasts, megakaryocytes, B cells, and T cells (Additional file 2: Fig. S1). These populations were then subjected to a targeted single-cell RNA-sequencing mutation analysis, covering *U2AF1*, *SF3B1*, *TET2*, *ASXL1*, *TP53*, and *DNMT3A*, the most frequently mutated genes in MDS [1, 2] (Additional file 2: Fig. S2A–C). After filtration, we obtained 3,549 single cells (Additional file 2: Fig. S3A–C). A total of eighteen mutations were detected from all donors. All mutations were reported in COSMIC database. Three mutations detected in both the HD and MDS groups were termed CH mutations while the remaining fifteen mutations only detected in MDS group were termed MDS mutations (Fig. 1A, Additional file 1: Table S2), although the border between CH related mutations and MDS/AML related mutations is still blurry [8]. The fraction of cells carrying CH mutations was similar to that carrying MDS mutations (Fig. 1B). Greater mutation number in the MDS group than that in the HD group was observed (Fig. 1C).

**Fig. 1:**
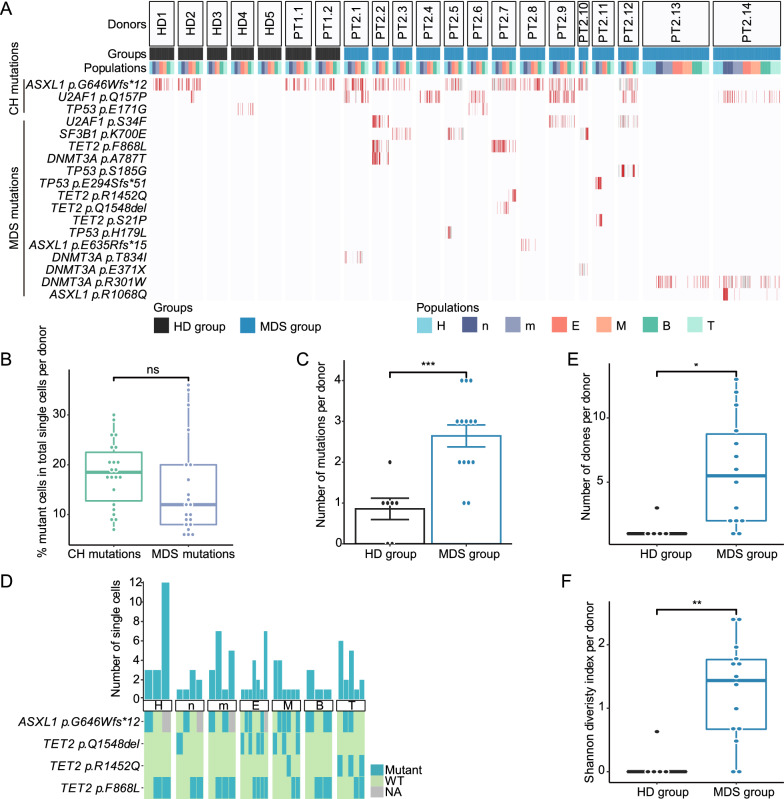
Comparison of mutations and clones between HD and MDS groups. **A** Data include all single cells analyzed by single-cell RNA-seq. Data of single cells in the HPCs (H), neutrophils (n), monocytes (m), erythroblasts (E), megakaryocytes (M), B cells (B), and T cells (T) populations from individual donors are shown in columns. Detected mutations are shown in rows. Mutations are shown in red. WT is shown as blank. When mutation sites had <10 reads, we could not determine whether mutations exist and data are shown in gray as NA (not available).** B** The percentage of mutant cells among the total single cells examined per donor. Data are presented as the medians with interquartile ranges. **C** Number of identified mutations per donor. Data are presented as the mean ± S.E. **D** The clonal analysis of PT2.7 is shown as an example. Eleven distinct clones were detected based on combinations of four mutations and some clones were detected in multiple populations. The upper panel shows the number of cells detected in a clone. Clones are identified with the combination of mutations. The lower panel shows the combinations of mutations detected in each clone. **E** Number of clones per donor in the HD group and MDS group. **F** Clonal diversity examined using the Shannon diversity index. Mann–Whitney *U* test was used in Fig. 1B, E, F, and two-sided Student’s *t* test was used in Fig. 1C. ns, *p *> 0.05; *, *p* < 0.05; **, *p* < 0.01; and ***, *p* < 0.001. **D**–**F** Wild type (WT) clones without mutations were excluded

Clones were defined as cells with identical combinations of mutations [9] (Fig. 1D). A total of eighty-nine clones were detected in all donors. CH clones were marked with CH mutations while MDS clones were marked with MDS mutations alone or both CH mutations and MDS mutations (Additional file 2: Fig. S4A). Greater clone number and clonal diversity in the MDS group than that in the HD group were observed (Fig. 1E, F), suggesting the accumulation of genetic lesions with more complexity during the progression to malignant diseases [10].

Most patients with MDS presented the mixture of multiple CH clones and MDS clones (Fig. 2A). The clone size of CH clones was significantly greater than that of MDS clones (Additional file 2: Fig. S4B), even within the MDS group (Fig. 2B), suggesting the acquisition of CH mutations occurs earlier than that of MDS mutations [11]. No statistical difference in the clone size of CH clones between HD and MDS groups was observed (Fig. 2C). Both CH mutant clones and MDS mutant clones were larger in clone size than double mutant clones from MDS group, suggesting that CH mutations do not provide a significant growth advantage to MDS mutations (Fig. 2D).

**Fig. 2:**
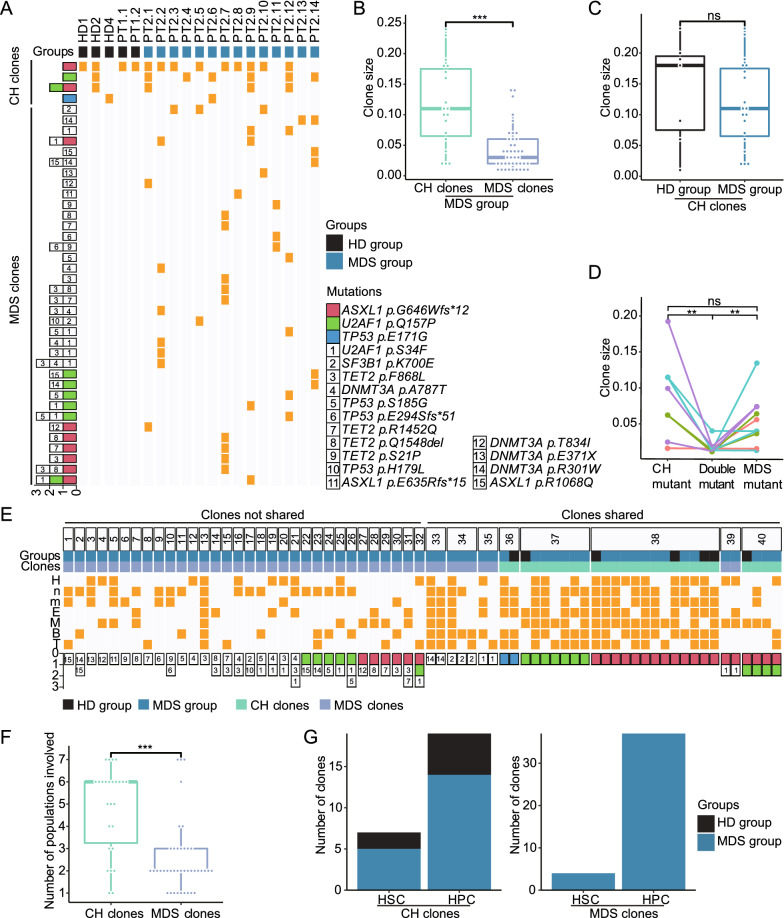
Donor and lineage distributions of CH clones and MDS clones. **A** Donor distributions of CH clones and MDS clones. Each column represents a donor. Each row represents a clone. The mutation composition per clone is shown on the far left. The presence of the clone in donors is shown as a yellow grid. Data of HD3 and HD5 were excluded since neither mutations nor clones were detected. **B** Comparison of the clone size between CH clones and MDS clones from the MDS group. **C** Comparison of the clone size of CH clones between the HD and MDS groups. **D** Comparison of the clone size among MDS clones marked with both CH mutations and MDS mutations (Double mutant clones), CH clones with the same CH mutations alone (CH mutant clones) and MDS clones with the same MDS mutations alone (MDS mutant clones) in eleven cases of four MDS patients. Clones from the same donors were connected with the line and shown in the same color. **E** Lineage distributions of CH clones and MDS clones. Each column represents one clone. The same clones were grouped under the same number. Clones not shared by two or more donors (Clones not shared) and clones shared by two or more donors (Clones shared) are separately shown. The presence of a clone in seven populations is shown as a yellow grid. The mutation composition per clone is shown on the bottom panel (see legends in part figure **A**). **F **Number of populations involved in CH clones and MDS clones. Each dot represents a clone. **G** Number of clones originating from HSCs and HPCs. (**A-G**) Mann–Whitney *U* test was used in all comparison analysis. ns, *p *> 0.05; **, *p* < 0.01; and ***, *p *< 0.001. Clones without any mutations and clones with uncertain mutations (NA) were excluded from these analyses

CH clones and MDS clones were detected in a variety of lineage combinations (Fig. 2E). The number of cell populations involved in CH clones was significantly greater than that in MDS clones (Fig. 2F). When the same clones were detected in all six lineage populations regardless of H population, they were considered to originate from HSCs, otherwise from HPCs. 27% CH clones were derived from HSCs while 10% MDS clones were derived from HSCs (Fig. 2G). Of note, exact HPC types could not be defined by our strategy. 10 out of 37 HPC-derived MDS clones were found in MDS-EB1 patients and 19 out of 37 HPC-derived MDS clones was found in MDS-EB2 and sAML patients. MDS with higher blasts seems to have more chance to have HPC origin (Additional file 1: Table S3). But the percentage of blasts was not related to clone distribution (Additional file 2: Fig. S4C). A small number of single cells such as 18–26 cells per population were examined in previous single-cell analysis of MDS/AML [12]. In two MDS patients (PT2.13 and PT2.14), more single cells were analyzed, confirming the HSC and HPC origins for most MDS clones.

The number of HSC-derived clones might be underestimated, because a limited number of cells per population was analyzed. Nevertheless, if not all, MDS appeared to be the mixture of CH clones and MDS clones originating from HSCs and HPCs.

## Supplementary Information

**Additional file 1: Table S1.** BM donors. **Table S2.** Comparison of mutations detected by targeted bulk DNA sequencing and targeted single cell RNA sequencing. **Table S3.** The number of HSC- and HPC-derived clones in individual donors. **Table S4.** Primers. **Table S5.** Barcode, index and adaptor sequences. **Table S6.** Targeted genes in DNA sequencing.

**Additional file 2: Figure S1.** Gating strategy for seven cell populations. FACS strategy used to isolate single cells from the seven populations: H, CD34^+^CD45^low^ cells; E, CD45^−^CD235a^+^CD71^+^ cells; M, CD45^−^CD235a^−^CD41^+^ cells; m, CD45^+^CD33^+^CD14^+^CD16^−^ cells; n, CD45^+^CD33^+^CD14^−^CD16^+^ cells; B, CD45^+^CD19^+^CD20^+^ cells; and T, CD45^+^CD3^+^ cells. The upper panels represent HD data, and the lower panels represent data from a patient with MDS. **Figure S2.** Targeted single-cell RNA-seq method. **A** Schematic diagram of the targeted single-cell RNA-seq method. Single H, n, m, E, M, B, and T were sorted by flow cytometry into a 96-well plate. One-step RT-PCR was performed with the first-round primer mix. The second round of PCR was performed with PCR primer mix 1 or mix 2. Finally, a sequencing library was constructed with p5 and p7 adaptors. **B** Frequency at which the *DNMT3A p.R301W *mutation was detected in 32 single JURKAT cells. The positive rate was 78.1%. **C** Electrophoretogram of the representative libraries from single cells and 20 cells from a patient with MDS. One pair of each primer (*U2AF1, SF3B1, ASXL1, TP53, TET2*, and *DNMT3A*) from the PCR primer mix (PCR primer mix 1 or PCR primer mix 2) was used for the second round of PCR, followed by library construction. PCR products were separated on a gel. Library construction performed without cells served as a negative control (neg). **Figure S3.** Qualification of single cell RNA-seq data. **A **The results of single-cell filtration. The number of qualified and unqualified single cells is shown for individual donors. Only qualified cell data were used in the analysis. **B **Coverage of each mutation in single cells after filtration. The total number of reads covering each mutation site in qualified single cells is shown as the coverage. The threshold of coverage was 10x and is marked by a horizontal dotted line. Data are presented as the medians with interquartile ranges. Data points that fell outside of the upper and lower whiskers were considered outliers and are not shown. **C **Variant allele frequency (VAF) of mutations after filtration. Each dot represents the VAF of each mutation from single cells of donors carrying this mutation after filtration. **Figure S4.** The clone landscape in all donors. **A **Data include all clones identified. Eighteen identified mutations were used to detect eighty-nine clones from all donors. Each column represents a clone. CH clones were identified with CH mutations. MDS clones were identified with MDS mutations regardless of the coexistence of CH mutations. The lineage distributions of clones are shown in orange in the upper panel. The composition of mutations in each clone is shown in the lower panel. The presence of mutations is shown in red. WT is shown as blank. Mutation sites without enough reads to determine genotypes are shown in gray as not available (NA). The bottom panel shows the clone size. Wild type (WT) clones without mutations were excluded. Data of HD3 and HD5 are not shown since neither mutations nor clones were detected. **B** Clone size of CH clones and MDS clones from all donors. ***, *p *< 0.001 (Mann–Whitney *U *test). **C** Clone size and number of populations involved in clones were compared between low blasts and high blasts groups. Fourteen patients with MDS were classified into low blasts (1%-9%) group (n = 8) mainly consisting of EB1 and high blasts (>9%) group (n = 6) mainly consisting of EB2. ns, *p* > 0.05 (Mann–Whitney *U *test).

## Data Availability

Supplementary information including Additional file 2: Figs. S1–S4 and Additional file 1: Tables S1–S6 are provided with the online version of this paper. The datasets analyzed during the current study are available from the corresponding author on reasonable request.

## Abbreviations

MDS: Myelodysplastic syndromes
BM: Bone marrow
CH: Clonal hematopoiesis
sAML: Secondary acute myeloid leukemia
HD: Healthy donor
HSC: Hematopoietic stem cell
HPC: Hematopoietic progenitor cell
RT-PCR: Reverse transcription-polymerase chain reaction
