# Supplementary material for: Myelodysplastic syndromes are multiclonal diseases derived from hematopoietic stem and progenitor cells
Source: Exp Hematol Oncol. 2022 May 16;11:28. doi: 10.1186/s40164-022-00280-3 (PMC9109331; doi:10.1186/s40164-022-00280-3)
Supplement: Supplementary file 1 — Additional file 1: Table S1. BM donors. Table S2. Comparison of mutations detected by targeted bulk DNA sequencing and targeted single cell RNA sequencing. Table S3. The number of HSC- and HPC-derived clones in individual donors. Table S4. Primers. Table S5. Barcode, index and adaptor sequences. Table S6. Targeted genes in DNA sequencing. [file 40164_2022_280_MOESM1_ESM.docx]

**Myelodysplastic syndromes are multiclonal diseases derived from hematopoietic stem and progenitor cells**

Bingqing Luo^1^, Fang Dong^1^, Tiejun Qin^2^, Qingyun Zhang^1^, Haitao Bai^1^, Jinhong Wang^1^, Yujiao Jia^1^, Shihui Ma^1^, Erlie Jiang^1^, Tao Cheng^1^, Zhijian Xiao^2^, & Hideo Ema^1^

**Supplementary Data**

**Supplementary Methods**

**Bone marrow cells**

BM cells were collected from patients with MDS and HDs after obtaining informed consent in compliance with the Declaration of Helsinki. All procedures were approved by the Ethics Committee, Institute of Hematology and Blood Diseases Hospital, Chinese Academy of Medical Sciences & Peking Union Medical College. The diagnosis of MDS was based on the 2016 revised criteria of the World Health Organization classification of myeloid neoplasms and acute leukemia. The donor information is shown in Supplementary Table S1.

**Flow cytometry and single-cell isolation**

BM cells were mixed with phosphate-buffered saline (PBS) at a 1:1 volume. The mixture was slowly overlaid onto an equal volume of Histopaque (Sigma) in a centrifuge tube (Corning). The tube was centrifuged at 2,500 rpm (1455 rcf/G) for 30 min at 20°C without breaking. The interface layer containing mononuclear cells was collected, mixed with the same volume of PBS, and centrifuged at 2,000 rpm (931 rcf/G) for 10 min at 20°C. After counting the cells, cells were centrifuged at 1,200rpm (335 rcf/G) for 5 min at 4°C. Cells were divided into two aliquots: one was stained with antibodies in the panel-1 for the H, E, and M populations. The other was stained with antibodies in the panel-2 for the n, m, B, and T populations. 7-AAD (Biolegend) or DAPI (4’,6-diamidino-2-phenylindole dihydrochloride, Sigma) was used to exclude dead cells. Cells were stained with antibodies on ice in the dark for 30 min and then sorted on a FACSAria III instrument (BD Biosciences) in single-cell sorting mode. Seven populations of cells were sorted into 96-well plates (MicroAmp™ Optical 96-Well Reaction Plate with Barcode, Thermo Fisher). Subsequent data analyses were performed with the FlowJo V.10 analysis software.

**Antibody panel-1**

| **Antigens** | **Conjugation** | **Clones** | **Suppliers** | **Dilution** |
| --- | --- | --- | --- | --- |
| CD34 | APC | 581 | BD Biosciences | 1:50 |
| CD45 | PE-Texas red | 2D1 | Biolegend | 1:100 |
| CD235a | FITC | 11E4B-7-6 | Beckman Coulter | 1:50 |
| CD71 | BV786 | M-A712 | BD Biosciences | 1:200 |
| CD41 | AF700 | HIP8 | Biolegend | 1:100 |

**Antibody panel-2**

| **Antigens** | **Conjugation** | **Clones** | **Suppliers** | **Dilution** |
| --- | --- | --- | --- | --- |
| CD45 | PE-Texas red | 2D1 | Biolegend | 1:100 |
| CD33 | FITC | HIM3-4 | Biolegend | 1:500 |
| CD14 | PE-CY7 | 63D3 | Biolegend | 1:200 |
| CD16 | BV650 | 3G8 | Biolegend | 1:50 |
| CD3 | BV510 | OKT4 | Biolegend | 1:50 |
| CD19 | PE | HIB19 | Biolegend | 1:50 |
| CD20 | BV785 | 2H7 | Biolegend | 1:200 |

**RT-PCR primer design and Targeted single-cell RNA sequencing**

We designed primers for two rounds of PCR. Reverse transcription-PCR (RT-PCR) consisted of 11 pairs of primers (first-round primer) for 6 genes and their specificity was verified by PrimerBlast in NCBI. For the second round of PCR, nested primers were used to amplify 300-500 bp amplicons: 20 and 6 pairs of primers were used together as second round primer mix 1 and mix 2, respectively. Forward primers consisted of the common sequence (ACACTCTTTCCCTACACGACGCTCTTCCGATCT) and a target-specific sequence. Reverse primers consisted of the common sequence (GTGACTGGAGTTCAGACGTGTGCTCTTCCGATCT), a barcode sequence (e.g., AACGTGAT), and target-specific reverse sequence. P5 and P7 primers used for library construction consisted of universal adaptors and a dual 6-base index. Supplementary Tables S4 and S5 show the sequences of all primers used.

Single cells were sorted into a 96-well plate (MicroAmp™ Optical 96-Well Reaction Plate with Barcode, Thermo Fisher). Each well contained 5 μl of 2X Reaction Mix, 0.5 μl of SuperScript™III RT/Platinum® Taq Mix (Invitrogen), 2 μl of DNA Suspension Buffer (Teknova), 0.2 μl of RNaseOUT™ Recombinant Ribonuclease Inhibitor (Thermo Fisher), and 2.5 μl of 0.18 μM first-round primer mix (Invitrogen). Reverse transcription was performed at 50 °C for 15 min and then incubated at 95 °C for 2 min for initialization, followed by 22 cycles of 95 °C for 15 sec and 60 °C for 4 min. PCR products were diluted 5-fold with nuclease-free water (UltraPure™ DNase/RNase-Free Distilled Water, Thermo Fisher). Next, 1 μl of diluted RT-PCR products was mixed with 5 μl of 2X KAPA HiFi HotStart readyMix (KAPA Biosystems), 2 μl of nuclease-free water, and 2 μl each of 1 μM second round primer mix 1 and mix 2. PCR was performed at 95 °C for 3 min; 30 cycles of 98 °C for 20 sec, 64 °C for 15 sec, and 72 °C for 30 sec; and 1 cycle of 72 °C for 1 min. PCR products from 32 or 48 single cells (3 μl of each) were pooled for PCR mixes to construct libraries. Subsequently, Ampure XP beads (Beckman Coulter) were used at a 1:1 bead to cDNA ratio to clean up excess primers, salts, and enzymes. During library construction, 3 μl of the reaction was mixed with 1.8 μl of nuclease-free water, 5 μl of 2X KAPA HiFi HotStart ReadyMix, 0.1 μl of P5 adaptor (20 μM) and 0.1 μl of P7 adaptor (20 μM). PCR was performed at 95 °C for 3 min; 15 cycles of 98 °C for 20 sec, 64 °C for 15 sec, and 72 °C for 30 sec; and 1 cycle of 72 °C for 1 min. Ampure XP beads were used at a 0.8:1 bead to cDNA ratio to clean up the final PCR products again. Libraries were quantified using Qubit (Thermo Fisher), and the size distribution was examined using an Agilent Bioanalyzer 2100 (Agilent). Sequencing was performed on an Illumina NovaSeq 6000 PE250/PE150 platform.

**Cell line**

JURKAT cells were obtained from the American Type Culture Collection (ATCC) and cultured in RPMI-1640 (Invitrogen) supplemented with 10% fetal calf serum (FCS, Thermo Fisher) and antibiotics. Thirty-two single JURKAT cells were sorted into a 96-well plate. The first round of PCR was performed as described above. For the second round of PCR, LDF1 and LDR1 primers (Supplementary Table S4) were used to detect the *DNMT3A p.R301W* mutation.

**Targeted bulk DNA sequencing of BM cells**

DNA was extracted from bulk BM mononuclear cells. Myeloid neoplasm genes were targeted using an Ion AmpliSeq^TM^ kit (Thermo Fisher Scientific) with a 137 Gene Panel (Supplementary Table S6). Automated library construction and chip loading were performed using an Ion Chef^TM^ system (Thermo Fisher Scientific). Sequencing was performed on an Ion Proton sequencer (Thermo Fisher Scientific). Mutation analysis was performed with Torrent Suite, Ion Reporter, and Variant Reporter software. Variants were individually assessed with COSMIC, dbSNP, Polyphen-2, 1000 Genomes, ESP 6500 database, and published literature to suggest their pathogenicity. Variants with > 1% population frequency were considered polymorphisms and removed. Thresholds of VAF for point mutations were set to 5% and 2% for insertions and deletions, respectively.

**Data analysis**

The quality of raw sequencing data was assessed using FastQC [1], and raw data were processed with fastp [2] to trim adaptors and remove low-quality bases. Sequencing data were divided into individual single-cell data using Cutadapt [3]. Reads were aligned to hg38 using STAR [4]. Alignment results were processed as described in GATK best practice workflows for RNAseq short variant discovery [5]. Mutations were called with HaplotypeCaller, and data from single cells were combined for each donor using CombineGVCFs. Hard filtration was implemented in VariantFiltration. Annotation was performed using annovar [6]. The minimum variant quality score was set to 30 with a minimum of 10 reads per variant per cell.

The following filtration criteria were used in order: (1) only nonsynonymous mutations in exon regions found in the COSMIC database or reported in bulk DNA sequence were selected; (2) mutations with > 1% population frequency in 1000 Genomes and ExAC databases were removed; (3) Variant allele frequency (VAF) was defined as the percentage of mutant reads in the total reads at each mutation site. The VAF < 5% was considered WT, except that the VAF < 20% was considered WT for the *ASXL1 p.G646Wfs*12*, *U2AF1 p.Q157P* and *U2AF1 p.S34F* mutation; (4) mutations detected in < 5% of all single cells from a donor were removed; (5) mutations were removed when no genotypes were obtained in > 40% of all single cells from a donor; (6) single cells were removed when no genotypes were obtained for > 50% of predicted mutations; and (7) mutations detected in < 5% of all single cells from a donor were removed again. We manually deleted the *ASXL1 p.G645Vfs*58*, *ASXL1 p.G645Wfs*12*, *ASXL1 p.G646Vfs*58* mutations which were considered as artefacts attributed to PCR errors. We also deleted the *TET2 p.Q1527X* and *TP53 p.P85L* mutations because they were frequently found in HDs, indistinguishable from single nucleotide polymorphisms.

Clones were defined as cells with identical combinations of mutations. To detect a clone, mutation(s) were detected in two or more single cells per donor. The clone size was defined as the ratio of the number of single cells with mutation(s) divided by the total number of single cells analyzed per donor. The dominant clone was defined as a clone with the largest clone size per donor.

**Statistical analysis and plotting**

Statistical analyses were performed using Two-sided Student’s *t* test, Mann–Whitney *U* test and Kruskal-Wallis test. The Shannon diversity index was calculated with the vegan package [7] in R to evaluate clonal diversity. Data were significantly different when the *P* value was < 0.05. Data were visualized with the ggplot2 [8], ComplexHeatmap [9] and igraph [10] packages in R.

**Further Discussion**

A number of CH clones and MDS clones were identified in this study. When we compared the clone size of double mutant clones and single mutant clones in the same patients, the latter was much greater than the former (Fig. 2D), suggesting that CH clones may not play a role in clonal expansion of MDS although CH clones likely play a role in a premalignant state [11]. Additional conditions may be required for CH clones to dominate one another [12]. Mouse model studies have recently suggested the interference of environmental factors in the initiation of clonal expansion of HSCs carrying CH mutations [13]. Further functional studies are warranted to understand the mechanisms underlying the evolution of CH clones into hematologic neoplasms.

The present study suggested that multiple MDS clones were responsible for MDS pathogenicity. However, we did not observe a large clonal expansion of MDS clones compared to CH clones. Clonal expansion might not have been detected in this study due to the limited numbers of targeted genes and analyzed cells. However, the pathogenicity of MDS clones detected cannot be denied merely based on clone size. A recent study has suggested that the clone size in MDS is not always associated with disease progression to sAML [14]. Some MDS clones may undergo a marked expansion by acquiring additional driver mutations and finally develop into sAML.

MDS-EB2 patients had more HPC-derived MDS clones than MDS-EB1 patients, suggesting that the HPC origin may have a higher risk to develop MDS with a higher percentage of blasts (Supplementary Table S3). This is in line with recent studies that MDS later progressing to sAML were propagated from HPCs rather than HSCs [15-17]. Further study may be warranted to investigate the mechanism.

**Table S1. BM donors.**

| **Donor ID** | **Age** | **Sex** | **Sequenced cells** | **Diagnosis** | **Treatment** | **Blasts in BM** |
| --- | --- | --- | --- | --- | --- | --- |
| HD1 | 39 | F | 24 $\times$ 7 |  |  |  |
| HD2 | 47 | M | 24 $\times$ 7 |  |  |  |
| HD3 | 45 | F | 24 $\times$ 7 |  |  |  |
| HD4 | 53 | M | 24 $\times$ 7 |  |  |  |
| HD5 | 31 | M | 24 $\times$ 7 |  |  |  |
| PT1.1 | 35 | M | 24 $\times$ 7 | AML-CR | HIA |  |
| PT1.2 | 37 | M | 24 $\times$ 7 | AML-CR | HIA |  |
| PT2.1 | 66 | M | 24 $\times$ 7 | MDS-RS | No treatment | 1.5% |
| PT2.2 | 46 | M | 24 $\times$ 6 + 8 | MDS-MLD | No treatment | 3% |
| PT2.3 | 58 | M | 24 $\times$ 7 | MDS-EB1 | No treatment | 3% (2% in PB) |
| PT2.4 | 51 | F | 24 $\times$ 7 | MDS-EB1 | EPO | 6% |
| PT2.5 | 60 | M | 24 $\times$ 7 | MDS-EB1 | decitabine | 8% |
| PT2.6 | 75 | M | 24 $\times$ 7 | MDS-EB1 | decitabine | 5% |
| PT2.7 | 44 | F | 24 $\times$ 7 | MDS-EB1 | EPO, danazol, azacitidine | 9% |
| PT2.8 | 47 | M | 24 $\times$ 7 | MDS-EB2 | No treatment | 11% |
| PT2.9 | 47 | M | 24 $\times$ 7 | MDS-EB2 | No treatment | 18% |
| PT2.10 | 33 | M | 10 $\times$ 7 | MDS-EB2 | No treatment | 13% |
| PT2.11 | 68 | M | 24 $\times$ 7 | MDS-EB2 | No treatment | 10% |
| PT2.12 | 65 | M | 24 $\times$ 7 | sAML | No treatment | 31% |
| PT2.13 | 37 | M | 64 $\times$ 6 + 88 | MDS-EB1 | No treatment | 5% |
| PT2.14 | 62 | F | 64 $\times$ 7 | MDS-EB2 | No treatment | 13.5% |

**Note**: The number of single cells analyzed is shown as the number of cells per population multiplied by the number of populations (Sequenced cells). 24 single cells from the six population (H, n, m, E, M, B) and 8 single T cells were analyzed in PT2.2. 64 single cells from the six populations (n, m, E, M, B, T) and 88 single H cells were analyzed in PT2.13.

HD, healthy donor; PT, patient; AML-CR, acute myeloid leukemia in complete remission; MDS, myelodysplastic syndromes; MDS-RS, MDS with ring sideroblasts; MDS-MLD, MDS with multilineage dysplasia; MDS-EB, MDS with excess blasts; MDS-EB1, BM blasts 5-9% or PB blasts 2-4% in the absence of Auer rods; MDS-EB2, BM blasts 10-19% or PB blasts 5-19% or the presence of Auer rods; sAML, secondary acute myeloid leukemia from MDS; EPO, erythropoietin; G-CSF, granulocyte colony-stimulating factor; HIA, homoharringtonine, idarubicin, and cytarabine.

**Table S2. Comparison of mutations detected by targeted bulk DNA sequencing and targeted single cell RNA sequencing.**

| **Donors** | **Only in DNA (VAF)** | **Both in DNA and RNA (VAF)** | **Only in RNA (mutant reads/ total reads in DNA data)** |
| --- | --- | --- | --- |
| HD1 |  |  | *ASXL1 p.G646Wfs*12* (**6/861**) |
| HD2 |  |  | *ASXL1 p.G646Wfs*12* **(8/1502)** *U2AF1 p.Q157P* **(0/0)** |
| HD3 |  |  |  |
| HD4 |  |  | *TP53 p.E171G* **(0/0)** |
| HD5 |  |  |  |
| PT1.1 |  |  | *ASXL1 p.G646Wfs*12* **(14/1140)** |
| PT1.2 |  |  | *ASXL1 p.G646Wfs*12* **(8/2360***)* |
| PT2.1 | *U2AF1 p.S34F* **(6.7%)** |  | *ASXL1 p.G646Wfs*12* **(0/20)** *U2AF1 p.Q157P* **(0/0)**  *DNMT3A p.T834I* **(0/0)** |
| PT2.2 |  | *U2AF1 p.S34F* **(35.1%)**  *DNMT3A p.A787T* **(36.6%)** | *ASXL1 p.G646Wfs*12* **(1/37)**  *TET2 p.F868L* **(945/1991)** |
| PT2.3 |  | *SF3B1 p.K700E* **(17.7%)** | *ASXL1 p.G646Wfs*12* **(1/17)** |
| PT2.4 |  |  | *U2AF1 p.Q157P* **(0/0)** |
| PT2.5 | *TP53 p.R280G* **(22.4%)** |  | *ASXL1 p.G646Wfs*12* **(7/1236)**  *SF3B1 p.K700E* **(0/0)**  *TP53 p.H179LP* **(3/1234)** |
| PT2.6 |  |  | *U2AF1 p.Q157P* **(0/0)**  *TP53 p.E171G* **(0/0)** |
| PT2.7 |  | *TET2 p.F868L* **(48.3%)** | *ASXL1 p.G646Wfs*12* **(44/3537)**  *TET2 p.R1452Q* **(1/3211***)*  *TET2 p.Q1548del* **(13/4199)** |
| PT2.8 |  | *ASXL1 p.E635Rfs*15* **(51.2%)** | *ASXL1 p.G646Wfs*12* **(0/0)** |
| PT2.9 |  | *U2AF1 p.S34F* **(46.9%)** | *ASXL1 p.G646Wfs*12* **(3/21)**  *U2AF1 p.Q157P* **(0/0)** |
| PT2.10 |  |  | *ASXL1 p.G646Wfs*12* **(3/237)**  *SF3B1 p.K700E* **(0/0)**  *DNMT3A p.E371X* **(0/0)** |
| PT2.11 |  |  | *TP53 p.E294Sfs*51* **(0/0)**  *TET2 p.S21P* **(0/0)** |
| PT2.12 | *TP53 p.S127T* **(86.4%)** |  | *ASXL1 p.G646Wfs*12* **(6/64)**  *U2AF1 p.Q157P* **(0/0)**  *U2AF1 p.S34F* **(0/0)**  *TP53 p.S185G* **(0/0)** |
| PT2.13 | *U2AF1 p.S34F* **(45%)** *ASXL1 p.Q708Rfs*17* **(41.3%)** |  | *DNMT3A p.R301W* **(2/3459)** |
| PT2.14 | *ASXL1 p.Q592** **(1.8%)** |  | *U2AF1 p.Q157P* **(0/0)**  *DNMT3A p.R301W* **(2/3998)**  *ASXL1 p.R1068Q* **(3/3442)** |

**Note:** Mutations were only detected by targeted bulk DNA sequencing (Only in DNA). Mutations were only detected by targeted single-cell RNA-sequencing (Only in RNA). Mutations were detected by both targeted bulk DNA and targeted single-cell RNA sequencing (Both in DNA and RNA). (0/0) indicates that sites were not covered by DNA seq analysis.

Some mutations detected by targeted single-cell RNA sequencing were not detected by targeted bulk DNA sequencing. There were three possible causes: (1) Some mutation sites were not covered with reads in bulk DNA sequencing; (2) The site of frequent mutation *ASXL1 p.G646Wfs*12* had too few mutant reads to be reported in bulk DNA sequencing; and (3) The mutation *TET2 p.F868L* from PT2.2 was considered as SNP and not reported as a mutation in bulk DNA sequence data at that time. Most additional mutations detected by single-cell RNA sequencing was not well covered by bulk DNA sequencing. DNA sequencing is considered as a gold standard for mutation analysis, but insufficient site coverage and amplicon bias are pervasive in targeted DNA sequencing [18, 19], causing false negative data.

Some mutations detected by targeted bulk DNA sequencing were not detected by targeted single RNA sequencing. There were three possible causes: (1) Mutations were detected but filtered out due to dissatisfying our criteria. The mutation *U2AF1 p.S34F* in PT2.1 and PT2.13 and the mutation *TP53 p.R280G* in PT2.5 were detected in scRNA-seq, but these mutations did not pass the filter criteria. Because their genotypes were obtained in < 40% of all single cells from a donor or detected in < 5% of all single cells per donor; (2) The mutation sites were not covered with sufficient number of reads. The mutation *ASXL1 p.Q708Rfs*17* in PT2.13 was not detected by scRNA-seq due to no reads coverage. The amplicon bias seemed to be more or less present in targeted amplicon sequencing; and (3) The mutation sites with sufficient number of reads coverage were detected as wild type. The mutation *TP53 p.S127T* was detected as WT in PT2.12 presumably because a large part of chromosome 17 was deleted in 12.6% cells when cells were examined by fluorescence immunofluorescence in situ hybridization. The mutation *ASXL1 p.Q592** was detected as WT in PT2.14. A possible reason was that we could not sort mutant cells in the case of a very low VAF (1.8%) by bulk DNA sequencing. RNA mutations usually represent DNA mutations well [20, 21], but some frameshift mutations may cause degradation of mRNA carrying this mutation and the normal allele might be more reverse-transcribed into cDNA [22], leading to false negative data.

**Table S3. The number of HSC- and HPC-derived clones in individual donors.**

| **Donors** | **Diagnosis** | **CH clones** | | **MDS clones** | |
| --- | --- | --- | --- | --- | --- |
|  |  | **HSC-derived** | **HPC-derived** | **HSC-derived** | **HPC-derived** |
| HD1 |  | 1 | 0 | 0 | 0 |
| HD2 |  | 1 | 2 | 0 | 0 |
| HD3 |  | 0 | 0 | 0 | 0 |
| HD4 |  | 0 | 1 | 0 | 0 |
| HD5 |  | 0 | 0 | 0 | 0 |
| PT1.1 | AML-CR | 0 | 1 | 0 | 0 |
| PT1.2 | AML-CR | 0 | 1 | 0 | 0 |
| PT2.1 | MDS-RS | 0 | 3 | 0 | 2 |
| PT2.2 | MDS-MLD | 0 | 1 | 0 | 6 |
| PT2.3 | MDS-EB1 | 0 | 1 | 1 | 0 |
| PT2.4 | MDS-EB1 | 1 | 0 | 0 | 0 |
| PT2.5 | MDS-EB1 | 0 | 1 | 0 | 2 |
| PT2.6 | MDS-EB1 | 0 | 2 | 0 | 0 |
| PT2.7 | MDS-EB1 | 1 | 0 | 1 | 8 |
| PT2.8 | MDS-EB2 | 0 | 1 | 0 | 1 |
| PT2.9 | MDS-EB2 | 2 | 1 | 0 | 4 |
| PT2.10 | MDS-EB2 | 0 | 1 | 0 | 2 |
| PT2.11 | MDS-EB2 | 0 | 0 | 0 | 3 |
| PT2.12 | sAML | 0 | 3 | 0 | 5 |
| PT2.13 | MDS-EB1 | 0 | 0 | 1 | 0 |
| PT2.14 | MDS-EB2 | 1 | 0 | 1 | 4 |
| Total |  | 7 | 19 | 4 | 37 |

Note: Clones without any mutations and clones with uncertain mutations (NA) were excluded from this table.

**Table S4. Primers.**

| **First round primers** | | |
| --- | --- | --- |
| **Gene** | **primer** | **sequence** |
| *U2AF1* | U1-F | ATCTTCGGCACCGAGAAAGA |
|  | U1-R | GGCTCAGAATCGCCCAGAT |
| *SF3B1* | S1-F | GAACACCACCAATGAGAAAGGC |
|  | S1-R | TACAATGGCCTTCAGTGCTCC |
| *TP53* | TP1-F | AAGTCTAGAGCCACCGTCCA |
|  | TP2-R | GTGCAGGCCAACTTGTTCAG |
| *ASXL1* | A1-F | GAGGCTAAGACTGACCCAGC |
|  | A1-R | TCATAGGAGGGCATGAGCCA |
|  | A2-F | ACAGCCCAAACCAGAATCCA |
|  | A2-R | TCCTGTGACATAGCACGGAC |
| *TET2* | T1-F | AGACCCGACTGCAACTGCT |
|  | T1-R | CTGAGCTTTGCTTGAAGTAAGC |
|  | T2-F | TTCTGCCACTACCACACCAC |
|  | T2-R | TCCTGTTCTTGAAAGCACCTGT |
|  | T3-F | AGAATCCACCTGCAAGCTGT |
|  | T3-R | GGATCTTGCTTCTGGCAAAC |
|  | T4-F | GGAAGTTTAAGCTGCTTGGG |
|  | T4-R | GGATGGGTGGTAGACTGAGC |
| *DNMT3A* | D1-F | CGATGACGAGCCAGAGTACG |
|  | D1-R | GCCCTGTAGCGATTCCATCA |
|  | D2-F | GGGAAGATCATGTACGTCGG |
|  | D2-R | ACTTTGTGTCGCTACCTCAGT |
| **Second round primers in the PCR mix1** | | |
| **gene** | **primer** | **sequence** |
| *U2AF1* | LU2-F | ACACTCTTTCCCTACACGACGCTCTTCCGATCTTGTGACAACCTGGGAGAC |
|  | LU2-R | GTGACTGGAGTTCAGACGTGTGCTCTTCCGATCT**AACGTGAT**TAGACCGAGAACGACGCT |
| *SF3B1* | LS1-F | ACACTCTTTCCCTACACGACGCTCTTCCGATCTCGTGGTCATTGAACCGCT |
|  | LS1-R | GTGACTGGAGTTCAGACGTGTGCTCTTCCGATCT**AACGTGAT**GCACAGCCCATAAGAATAGC |
|  | LS3-F | ACACTCTTTCCCTACACGACGCTCTTCCGATCTGCACAGGATGGCTTTGGAT |
|  | LS3-R | GTGACTGGAGTTCAGACGTGTGCTCTTCCGATCT**AACGTGAT**GTATGGTTTGACTCGTTTGCC |
| *TP53* | LTP1-F | ACACTCTTTCCCTACACGACGCTCTTCCGATCTCCAGGTCCAGATGAAGCTC |
|  | LTP1-R | GTGACTGGAGTTCAGACGTGTGCTCTTCCGATCT**AACGTGAT**CTCCACACGCAAATTTCCTTC |
| *ASXL1* | LA2-F | ACACTCTTTCCCTACACGACGCTCTTCCGATCTCTAGGAGAGAGGACCTG |
|  | LA2-R | GTGACTGGAGTTCAGACGTGTGCTCTTCCGATCT**AACGTGAT**AATCAGTCGGTGAGGAT |
|  | LA4-F | ACACTCTTTCCCTACACGACGCTCTTCCGATCTGGTCTAGATCCTCTTGACAGC |
|  | LA4-R | GTGACTGGAGTTCAGACGTGTGCTCTTCCGATCT**AACGTGAT**TCCACTGAGGACCCAGGCAT |
|  | LA5-F | ACACTCTTTCCCTACACGACGCTCTTCCGATCTAGTTGCTGCAGGGTAGCTT |
|  | LA5-R | GTGACTGGAGTTCAGACGTGTGCTCTTCCGATCT**AACGTGAT**GAGAACTGCTCTTTGGAATCC |
| *TET2* | LT1-F | ACACTCTTTCCCTACACGACGCTCTTCCGATCTAAGCAGGAGATGGGCTCA |
|  | LT1-R | GTGACTGGAGTTCAGACGTGTGCTCTTCCGATCT**AACGTGAT**GCCACTTGGTGTCTCCATT |
|  | LT2-F | ACACTCTTTCCCTACACGACGCTCTTCCGATCTTGAGATCACTCACCCATCG |
|  | LT2-R | GTGACTGGAGTTCAGACGTGTGCTCTTCCGATCT**AACGTGAT**ATACCGTTCAGAGCTGCCA |
|  | LT3-F | ACACTCTTTCCCTACACGACGCTCTTCCGATCTAAGCACTCTGAATGGTGGAG |
|  | LT3-R | GTGACTGGAGTTCAGACGTGTGCTCTTCCGATCT**AACGTGAT**GGCCTTCAATTCAATCCATC |
|  | LT4-F | ACACTCTTTCCCTACACGACGCTCTTCCGATCTAAGCCTCATAAACAGGCA |
|  | LT4-R | GTGACTGGAGTTCAGACGTGTGCTCTTCCGATCT**AACGTGAT**TTCAGGATGTGTAGTCTG |
|  | LT5-F | ACACTCTTTCCCTACACGACGCTCTTCCGATCTGCCAAGTCGTTATTTGAC |
|  | LT5-R | GTGACTGGAGTTCAGACGTGTGCTCTTCCGATCT**AACGTGAT**GCTTTACCCTTCTGTCCA |
|  | LT6-F | ACACTCTTTCCCTACACGACGCTCTTCCGATCTGATGTCCTATTGCTAAGTGGG |
|  | LT6-R | GTGACTGGAGTTCAGACGTGTGCTCTTCCGATCT**AACGTGAT**AGGCGCAAGTTCTCTCTTC |
|  | LT7-F | ACACTCTTTCCCTACACGACGCTCTTCCGATCTGAGGAAGAGAAACTGGAGTC |
|  | LT7-R | GTGACTGGAGTTCAGACGTGTGCTCTTCCGATCT**AACGTGAT**GTATAAAGGCAGAACGTG |
|  | LT8-F | ACACTCTTTCCCTACACGACGCTCTTCCGATCTGGAAGCTCAGGAGGAGAAA |
|  | LT8-R | GTGACTGGAGTTCAGACGTGTGCTCTTCCGATCT**AACGTGAT**TGAGGGTGATGTGGCTGCT |
|  | LT9-F | ACACTCTTTCCCTACACGACGCTCTTCCGATCTGTCTGTCAACTCTTATTCTGC |
|  | LT9-R | GTGACTGGAGTTCAGACGTGTGCTCTTCCGATCT**AACGTGAT**GCAGTTGTCCACTGATAGGT |
| *DNMT3A* | LD1-F | ACACTCTTTCCCTACACGACGCTCTTCCGATCTCTTTGGCATTGGGGAGCT |
|  | LD1-R | GTGACTGGAGTTCAGACGTGTGCTCTTCCGATCT**AACGTGAT**TCACTCTCATCGCTGTCGT |
|  | LD2-F | ACACTCTTTCCCTACACGACGCTCTTCCGATCTAACTGCAAGAACTGCTTTCTG |
|  | LD2-R | GTGACTGGAGTTCAGACGTGTGCTCTTCCGATCT**AACGTGAT**GCTTCCTCTTCTCAGCT |
|  | LD3-F | ACACTCTTTCCCTACACGACGCTCTTCCGATCTACACAGAAGCATATCCAGGAGT |
|  | LD3-R | GTGACTGGAGTTCAGACGTGTGCTCTTCCGATCT**AACGTGAT**TTGGCATCAATCATCACAGG |
|  | LD4-F | ACACTCTTTCCCTACACGACGCTCTTCCGATCTTATGAACAGGCCGTTGGCA |
|  | LD4-R | GTGACTGGAGTTCAGACGTGTGCTCTTCCGATCT**AACGTGAT**CCATGTCCCTTACACACA |
| **Second round primers in the PCR mix2** | | |
| **gene** | **primer** | **sequence** |
| *U2AF1* | LU1-F | ACACTCTTTCCCTACACGACGCTCTTCCGATCTGACAAAGTCAACTGTTC |
|  | LU1-R | GTGACTGGAGTTCAGACGTGTGCTCTTCCGATCT**AACGTGAT**GTTCATCTCCTCTACTTCCC |
| *SF3B1* | LS2-F | ACACTCTTTCCCTACACGACGCTCTTCCGATCTCTGTGCCATCTTGCCA |
|  | LS2-R | GTGACTGGAGTTCAGACGTGTGCTCTTCCGATCT**AACGTGAT**CATTTCCTCATCAGGAGAC |
| *ASXL1* | LA1-F | ACACTCTTTCCCTACACGACGCTCTTCCGATCTGAAAGTGTTCACACCGA |
|  | LA1-R | GTGACTGGAGTTCAGACGTGTGCTCTTCCGATCT**AACGTGAT**ATCACCACTGCTGCTGCCTCT |
| *TP53* | LTP2-F | ACACTCTTTCCCTACACGACGCTCTTCCGATCTATGAACCGGAGGCCCAT |
|  | LTP2-R | GTGACTGGAGTTCAGACGTGTGCTCTTCCGATCT**AACGTGAT**AACATCTCGAAGCGCTCA |
| *DNMT3A* | LD5-F | ACACTCTTTCCCTACACGACGCTCTTCCGATCTCTCTTTGAGAATGTGGTGG |
|  | LD5-R | GTGACTGGAGTTCAGACGTGTGCTCTTCCGATCT**AACGTGAT**CGAACTTAGCACCTGTGAAGAAGAT |
| *TET2* | LT10-F | ACACTCTTTCCCTACACGACGCTCTTCCGATCTGAGACTCATAATGTCC |
|  | LT10-R | GTGACTGGAGTTCAGACGTGTGCTCTTCCGATCT**AACGTGAT**CGAACTTATTATGGAGTTTGACCTC |

**Note**: The primer sequences are shown with Barcode 1 marked in bold in this list. Barcode1 can be replaced with one of Barcode 2-48 for the other primers in Supplementary Table S5.

**Table S5. Barcode, index and adaptor sequences.**

| **Barcode** | | | |
| --- | --- | --- | --- |
| **name** | **sequence** | **name** | **sequence** |
| Barcode1 | AACGTGAT | Barcode25 | AGCAGGAA |
| Barcode2 | AAACATCG | Barcode26 | AGTCACTA |
| Barcode3 | ATGCCTAA | Barcode27 | ATCCTGTA |
| Barcode4 | AGTGGTCA | Barcode28 | ATTGAGGA |
| Barcode5 | ACCACTGT | Barcode29 | CAACCACA |
| Barcode6 | ACATTGGC | Barcode30 | CAAGACTA |
| Barcode7 | CAGATCTG | Barcode31 | CAATGGAA |
| Barcode8 | CATCAAGT | Barcode32 | CACTTCGA |
| Barcode9 | CGCTGATC | Barcode33 | CATACCAA |
| Barcode10 | ACAAGCTA | Barcode34 | CCAGTTCA |
| Barcode11 | CTGTAGCC | Barcode35 | CCGAAGTA |
| Barcode12 | AGTACAAG | Barcode36 | CCGTGAGA |
| Barcode13 | AACAACCA | Barcode37 | CCTCCTGA |
| Barcode14 | AACCGAGA | Barcode38 | CGAACTTA |
| Barcode15 | AACGCTTA | Barcode39 | CGACTGGA |
| Barcode16 | AAGACGGA | Barcode40 | CGCATACA |
| Barcode17 | AAGGTACA | Barcode41 | CTCAATGA |
| Barcode18 | ACACAGAA | Barcode42 | CTGAGCCA |
| Barcode19 | ACAGCAGA | Barcode43 | CTGGCATA |
| Barcode20 | ACCTCCAA | Barcode44 | GAATCTGA |
| Barcode21 | ACGCTCGA | Barcode45 | GACTAGTA |
| Barcode22 | ACGTATCA | Barcode46 | GAGCTGAA |
| Barcode23 | AGAGTCAA | Barcode47 | GATAGACA |
| Barcode24 | AGATCGCA | Barcode48 | GCCACATA |
| **p5-index** | | | |
| **name** | **sequence** | | |
| Index5.1 | GCTCAT | | |
| Index5.2 | GTGTAT | | |
| Index5.3 | GAATGC | | |
| Index5.4 | AGAGTA | | |
| Index5.5 | AGTCCA | | |
| **P7-index** | | | |
| **name** | **sequence** | **name** | **sequence** |
| Index7.1 | ATCACG | Index7.25 | ACTGAT |
| Index7.2 | CGATGT | Index7.26 | TGAAGC |
| Index7.3 | TTAGGC | Index7.27 | ATTCCT |
| Index7.4 | TGACCA | Index7.28 | CAAAAG |
| Index7.5 | ACAGTG | Index7.29 | CAACTA |
| Index7.6 | GCCAAT | Index7.30 | CACCGG |
| Index7.7 | CAGATC | Index7.31 | CACGAT |
| Index7.8 | ACTTGA | Index7.32 | CACTCA |
| Index7.9 | GATCAG | Index7.33 | CAGGCG |
| Index7.10 | TAGCTT | Index7.34 | CATGGC |
| Index7.11 | GGCTAC | Index7.35 | CATTTT |
| Index7.12 | CTTGTA | Index7.36 | CCAACA |
| Index7.13 | AGTCAA | Index7.37 | CGGAAT |
| Index7.14 | AGTTCC | Index7.38 | CTAGCT |
| Index7.15 | ATGTCA | Index7.39 | CTATAC |
| Index7.16 | CCGTCC | Index7.40 | CTCAGA |
| Index7.17 | GTAGAG | Index7.41 | GACGAC |
| Index7.18 | GTCCGC | Index7.42 | TAATCG |
| Index7.19 | GTGAAA | Index7.43 | TACAGC |
| Index7.20 | GTGGCC | Index7.44 | TATAAT |
| Index7.21 | GTTTCG | Index7.45 | TCATTC |
| Index7.22 | CGTACG | Index7.46 | TCCCGA |
| Index7.23 | GAGTGG | Index7.47 | TCGAAG |
| Index7.24 | GGTAGC | Index7.48 | TCGGCA |
| **p5/p7 adaptors** | | | |
| **name** | **sequence** | | |
| p5 adaptor | AATGATACGGCGACCACCGAGATCTACAC**GCTCAT**TCTTTCCCTACACGACGCTCTTCCGATCT | | |
| p7 adaptor | CAAGCAGAAGACGGCATACGAGAT**CGTGAT**GTGACTGGAGTTC | | |

**Note**: p5 adaptor is shown with index 5.1 (in bold) which can be replaced with one of index5.2-5.5. P7 adaptor is shown with index 7.1 (in bold) which can be replaced with one of index7.2-7.48.

**Table S6. Targeted genes in DNA sequencing.**

| **Genes** | **Covered regions** | **Genes** | **Covered regions** | **Genes** | **Covered regions** | **Genes** | **Covered regions** |
| --- | --- | --- | --- | --- | --- | --- | --- |
| *ABL1* | CDS | *CSNK1A1* | Exon2-4 | *KLF2* | Exon1-3 | *RUNX1* | CDS |
| *ANKRD26* | Exon1/5’UTR | *CUX1* | CDS | *KMT2A* | CDS | *SETBP1* | CDS |
| *ARID1A* | CDS | *CXCR4* | CDS | *KMT2D* | CDS | *SETD2* | CDS |
| *ASXL1* | CDS | *DDX3X* | CDS | *KRAS* | CDS | *SF1* | CDS |
| *ASXL2* | CDS | *DDX41* | CDS | *MAP2K1* | Exon2-3 | *SF3B1* | CDS |
| *ATG2B* | CDS | *DIS3* | CDS | *MAPK1* | CDS | *SH2B3* | CDS |
| *ATM* | CDS | *DNM2* | Exon8/13/16/18/20 | *MAK* | CDS | *SMC1A* | CDS |
| *B2M* | CDS | *DNMT3A* | CDS | *MED12* | CDS | *SMC3* | CDS |
| *BCL2* | Exon2 | *DNMT3B* | CDS | *MEF2B* | Exon2-3 | *SPEN* | Exon11 |
| *BCL6* | 5’UTR | *EED* | CDS | *MPL* | CDS | *SRP72* | CDS |
| *BCOR* | CDS | *EGR1* | CDS | *MYC* | CDS | *SRSF2* | CDS |
| *BCORL1* | CDS | *EP300* | CDS | *MYD88* | CDS | *STAG2* | CDS |
| *BIRC3* | CDS | *ETNK1* | CDS | *NF1* | CDS | *STAT3* | CDS |
| *BRAF* | CDS | *ETV6* | CDS | *NOTCH1* | CDS | *STAT5B* | Exon11/13-18 |
| *BRINP3* | CDS | *EZH2* | CDS | *NOTCH2* | CDS | *SUZ12* | CDS |
| *BTK* | Exon5/11/14-19 | *FAM46C* | CDS | *NPM1* | CDS | *TAL1* | Exon3 |
| *CALR* | CDS | *FAT1* | CDS | *NRAS* | CDS | *TCF3* | Exon6/15/17 |
| *CARD11* | CDS | *FBXW7* | CDS | *NT5C2* | Exon9-16 | *TERT* | CDS |
| *CASP8* | Exon10 | *FGFR3* | CDS | *PAX5* | CDS | *TET2* | CDS |
| *CBL* | CDS | *FLT3* | CDS | *PDGFRB* | Exon18 | *TNFAIP3* | CDS |
| *CCND1* | CDS | *GATA1* | CDS | *PHF6* | CDS | *TNFRSF14* | Exon1-6 |
| *CCND2* | Exon4-5 | *GATA2* | CDS | *PIGA* | CDS | *TP53* | CDS |
| *CCND3* | CDS | *GATA3* | CDS | *PLCG1* | CDS | *TPMT* | CDS |
| *CCR4* | CDS | *GNA13* | Exon1-4 | *PLCG2* | CDS | *TRAF3* | CDS |
| *CD28* | Exon4 | *ID3* | CDS | *PPM1D* | CDS | *U2AF1* | CDS |
| *CD58* | Exon2-3 | *IDH1* | CDS | *PRDM1* | CDS | *USP7* | CDS |
| *CD79B* | CDS | *IDH2* | CDS | *PRKCB* | CDS | *WHSC1* | CDS |
| *CDC25C* | Exon8 | *IKZF1* | CDS | *PRPS1* | CDS | *WT1* | CDS |
| *CDKN1B* | CDS | *IL7R* | Exon5-6 | *PTEN* | CDS | *XPO1* | CDS |
| *CDKN2A* | CDS | *IRF4* | CDS | *PTPN11* | CDS | *ZBTB7A* | CDS |
| *CEBPA* | CDS | *JAK1* | CDS | *RAD21* | CDS | *ZMYM3* | CDS |
| *CNOT3* | Exon2-5 | *JAK2* | CDS | *RBBP6* | CDS | *ZRSR2* | CDS |
| *CREBBP* | CDS | *JAK3* | CDS | *RELN* | CDS |  |  |
| *CRLF2* | Exon6 | *KDM6A* | CDS | *RHOA* | Exon2-5 |  |  |
| *CSF3R* | CDS | *KIT* | CDS | *RPL10* | Exon5 |  |  |

**Figure legends**

**Figure S1. Gating strategy for seven cell populations.** FACS strategy used to isolate single cells from the seven populations: H, CD34+CD45low cells; E, CD45-CD235a+CD71+ cells; M, CD45-CD235a-CD41+ cells; m, CD45+CD33+CD14+CD16- cells; n, CD45+CD33+CD14-CD16+ cells; B, CD45+CD19+CD20+ cells; and T, CD45+CD3+ cells. The upper panels represent HD data, and the lower panels represent data from a patient with MDS.

**Figure S2. Targeted single-cell RNA-seq method**. **A** Schematic diagram of the targeted single-cell RNA-seq method. Single H, n, m, E, M, B, and T were sorted by flow cytometry into a 96-well plate. One-step RT-PCR was performed with the first-round primer mix. The second round of PCR was performed with PCR primer mix 1 or mix 2. Finally, a sequencing library was constructed with p5 and p7 adaptors. **B** Frequency at which the *DNMT3A p.R301W* mutation was detected in 32 single JURKAT cells. The positive rate was 78.1%. **C** Electrophoretogram of the representative libraries from single cells and 20 cells from a patient with MDS. One pair of each primer (*U2AF1, SF3B1, ASXL1, TP53, TET2*, and *DNMT3A*) from the PCR primer mix (PCR primer mix 1 or PCR primer mix 2) was used for the second round of PCR, followed by library construction. PCR products were separated on a gel. Library construction performed without cells served as a negative control (neg).

**Figure S3. Qualification of single cell RNA-seq data. A** The results of single-cell filtration. The number of qualified and unqualified single cells are shown for individual donors. Only qualified cell data were used in the analysis. **B** Coverage of each mutation in single cells after filtration. The total number of reads covering each mutation site in qualified single cells is shown as the coverage. The threshold of coverage was 10x and was marked by a horizontal dotted line. Data are presented as the medians with interquartile ranges. Data points that fell outside of the upper and lower whiskers were considered outliers and are not shown. **C** Variant allele frequency (VAF) of mutations after filtration. Each dot represents the VAF of each mutation from single cells of donors carrying this mutation after filtration.

**Figure S4. The clone landscape in all donors. A** Data include all clones identified. Eighteen identified mutations were used to detect eighty-nine clones from all donors. Each column represents a clone. CH clones were identified with CH mutations. MDS clones were identified with MDS mutations regardless of the coexistence of CH mutations. The lineage distributions of clones are shown in orange in the upper panel. The composition of mutations in each clone is shown in the lower panel. The presence of mutations is shown in red. WT is shown as blank. Mutation sites without enough reads to determine genotypes were shown in gray as not available (NA). The bottom panel shows the clone size. Wild type (WT) clones without mutations were excluded. Data of HD3 and HD5 are not shown since neither mutations nor clones were detected. **B** Clone size of CH clones and MDS clones from all donors. ***, *p* < 0.001 (Mann–Whitney *U* test). **C** Clone size and number of populations involved in clones were compared between low blasts and high blasts groups. Fourteen patients with MDS were classified into low blasts (1%-9%) group (n = 8) mainly consisting of EB1 and high blasts (>9%) group (n = 6) mainly consisting of EB2. ns, p > 0.05 (Mann–Whitney *U* test).

**Supplementary References**

1. de Sena Brandine G , Smith AD. Falco: high-speed FastQC emulation for quality control of sequencing data. F1000Res. 2019;8:1874.

2. Chen S, Zhou Y, Chen Y , Gu J. fastp: an ultra-fast all-in-one FASTQ preprocessor. Bioinformatics. 2018;34(17):i884-i890.

3. Martin M. Cutadapt Removes Adapter Sequences From High-Throughput Sequencing Reads. EMBnet.journal.

4. Dobin A, Davis CA, Schlesinger F, Drenkow J, Zaleski C, Jha S, et al. STAR: ultrafast universal RNA-seq aligner. Bioinformatics. 2013;29(1):15-21.

5. Poplin R, Ruano-Rubio V, DePristo MA, Fennell TJ, Carneiro MO, Van der Auwera GA, et al. Scaling accurate genetic variant discovery to tens of thousands of samples. bioRxiv. 2018.

6. Wang K, Li M , Hakonarson H. ANNOVAR: functional annotation of genetic variants from high-throughput sequencing data. Nucleic Acids Res. 2010;38(16):e164.

7. Oksanen J, Blanchet FG, Friendly M, Kindt R, Legendre P, McGlinn D, et al., *vegan: Community Ecology Package*. 2020.

8. Wickham H. ggplot2: Elegant Graphics for Data Analysis. Springer-Verlag New York. 2016.

9. Gu Z, Eils R , Schlesner M. Complex heatmaps reveal patterns and correlations in multidimensional genomic data. Bioinformatics. 2016;32(18):2847-9.

10. Csardi G , Nepusz T. The igraph software package for complex network research. InterJournal. 2006;Complex Systems:1695.

11. Jaiswal S, Fontanillas P, Flannick J, Manning A, Grauman PV, Mar BG, et al. Age-related clonal hematopoiesis associated with adverse outcomes. N Engl J Med. 2014;371(26):2488-98.

12. Zhang X, Su J, Jeong M, Ko M, Huang Y, Park HJ, et al. DNMT3A and TET2 compete and cooperate to repress lineage-specific transcription factors in hematopoietic stem cells. Nat Genet. 2016;48(9):1014-23.

13. Hormaechea-Agulla D, Matatall KA, Le DT, Kain B, Long X, Kus P, et al. Chronic infection drives Dnmt3a-loss-of-function clonal hematopoiesis via IFNγ signaling. Cell Stem Cell. 2021.

14. Chen J, Kao YR, Sun D, Todorova TI, Reynolds D, Narayanagari SR, et al. Myelodysplastic syndrome progression to acute myeloid leukemia at the stem cell level. Nat Med. 2019;25(1):103-110.

15. Woll PS, Kjallquist U, Chowdhury O, Doolittle H, Wedge DC, Thongjuea S, et al. Myelodysplastic syndromes are propagated by rare and distinct human cancer stem cells in vivo. Cancer Cell. 2014;25(6):794-808.

16. Mian SA, Rouault-Pierre K, Smith AE, Seidl T, Pizzitola I, Kizilors A, et al. SF3B1 mutant MDS-initiating cells may arise from the haematopoietic stem cell compartment. Nat Commun. 2015;6:10004.

17. Rouault-Pierre K, Smith AE, Mian SA, Pizzitola I, Kulasekararaj AG, Mufti GJ, et al. Myelodysplastic syndrome can propagate from the multipotent progenitor compartment. Haematologica. 2017;102(1):e7-e10.

18. Benjamini Y , Speed TP. Summarizing and correcting the GC content bias in high-throughput sequencing. Nucleic Acids Res. 2012;40(10):e72.

19. Morita K, Wang F, Jahn K, Hu T, Tanaka T, Sasaki Y, et al. Clonal evolution of acute myeloid leukemia revealed by high-throughput single-cell genomics. Nat Commun. 2020;11(1):5327.

20. Poirion O, Zhu X, Ching T , Garmire LX. Using single nucleotide variations in single-cell RNA-seq to identify subpopulations and genotype-phenotype linkage. Nat Commun. 2018;9(1):4892.

21. Castle JC, Loewer M, Boegel S, Tadmor AD, Boisguerin V, de Graaf J, et al. Mutated tumor alleles are expressed according to their DNA frequency. Sci Rep. 2014;4:4743.

22. Kaya C, Dorsaint P, Mercurio S, Campbell AM, Wha Eng K, Nikiforova MN, et al. Limitations of Detecting Genetic Variants from the RNA-Seq Data in Tissue and FNA Samples. Thyroid. 2020.
