# Supplementary figures and images for: Myelodysplastic syndromes are multiclonal diseases derived from hematopoietic stem and progenitor cells
Source: Exp Hematol Oncol. 2022 May 16;11:28. doi: 10.1186/s40164-022-00280-3 (PMC9109331; doi:10.1186/s40164-022-00280-3)

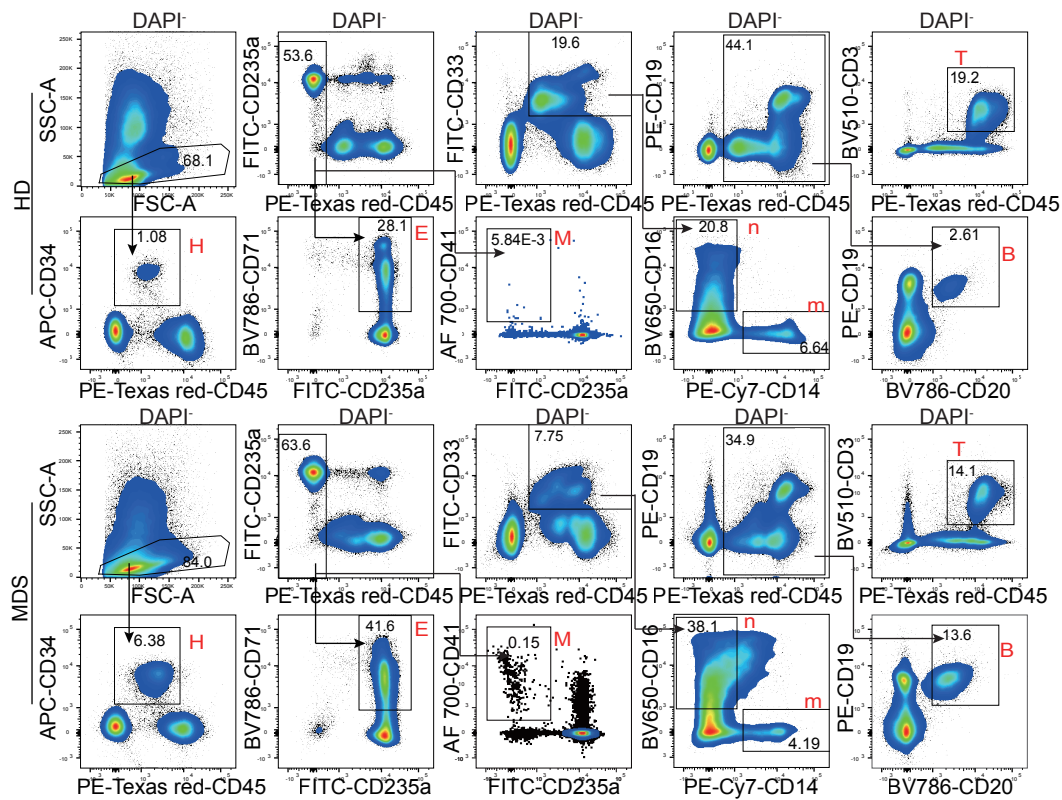

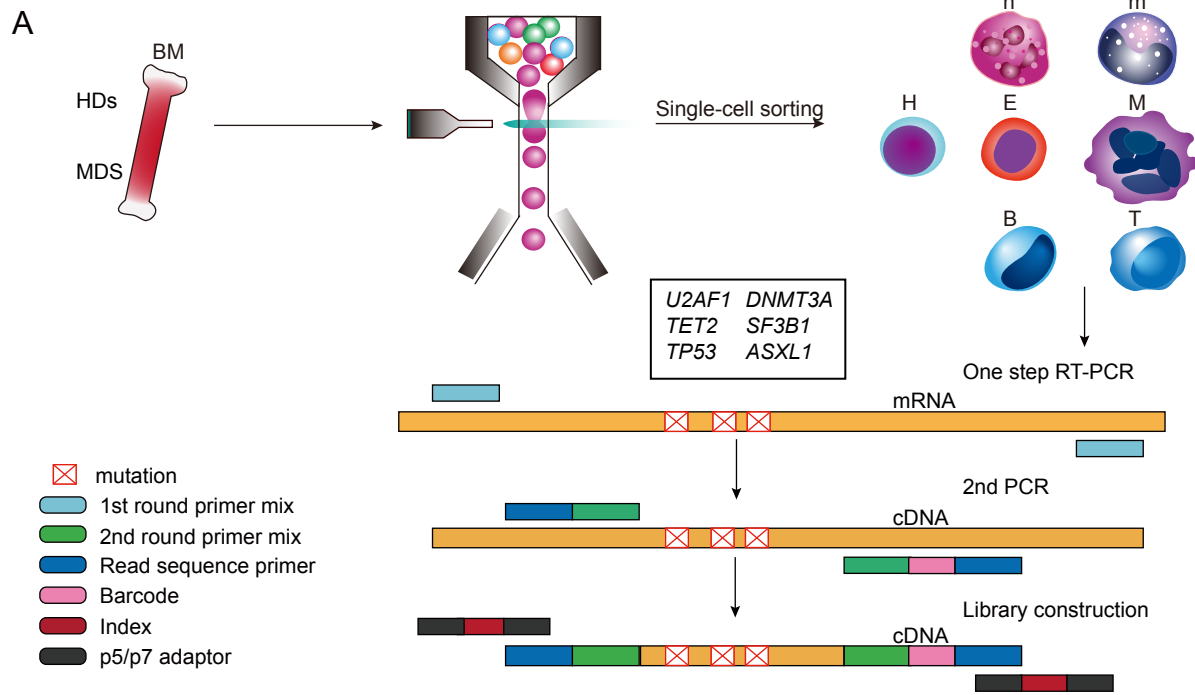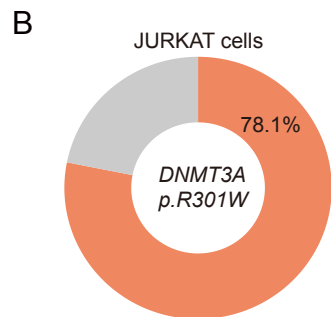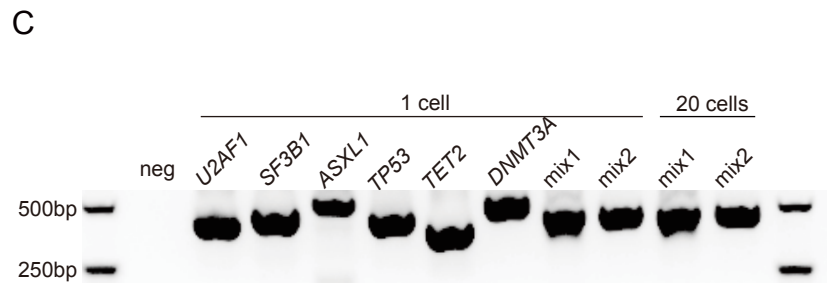

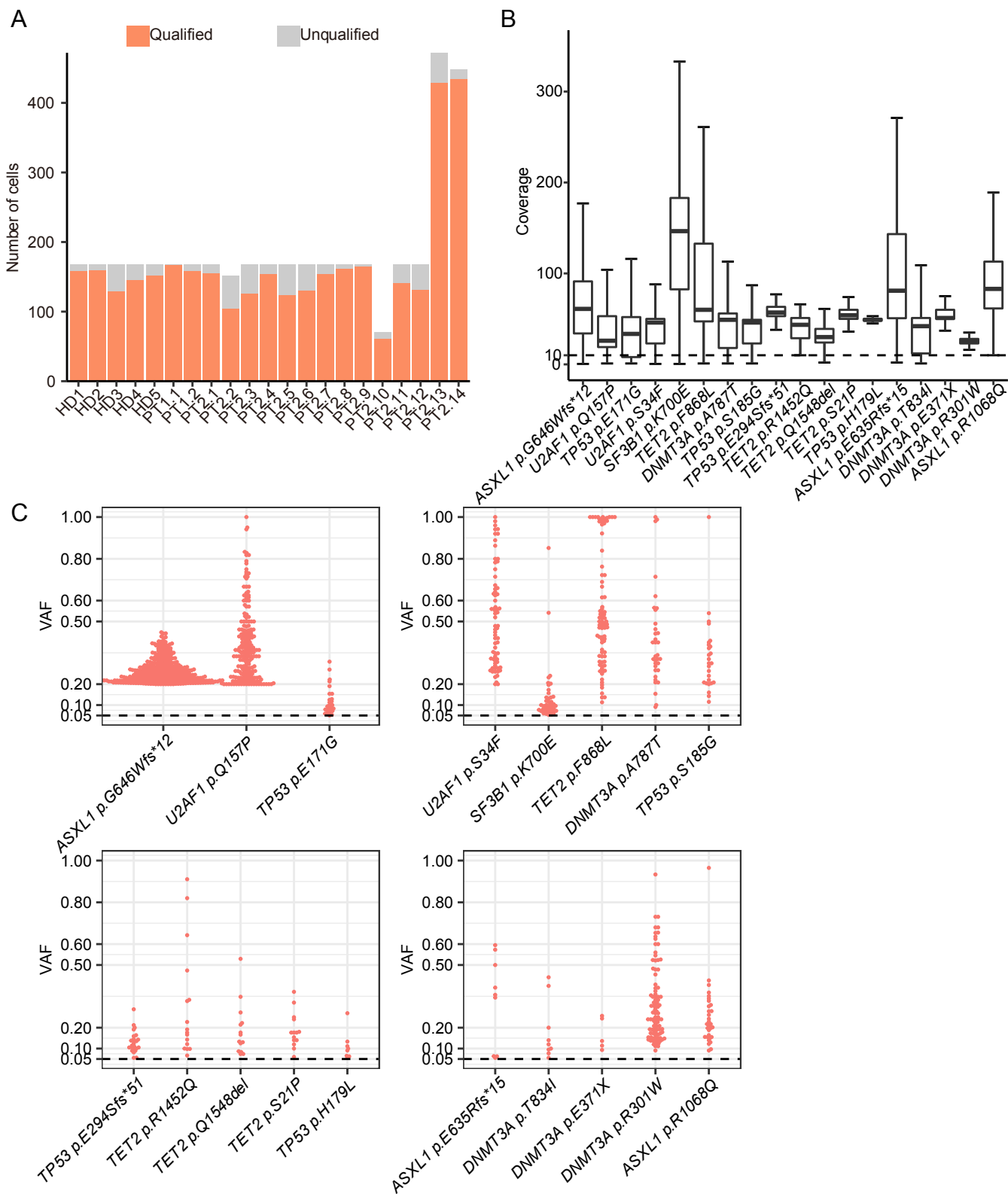

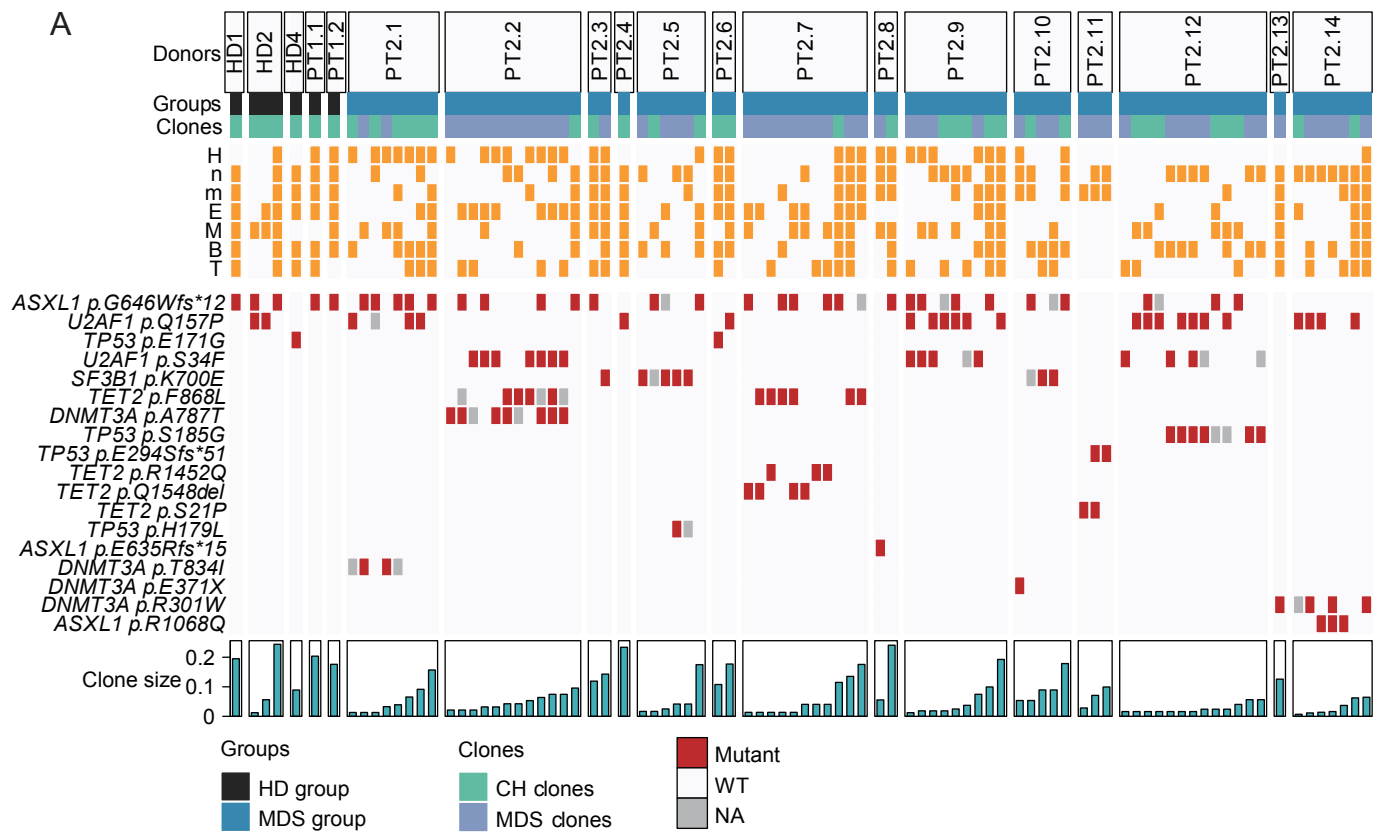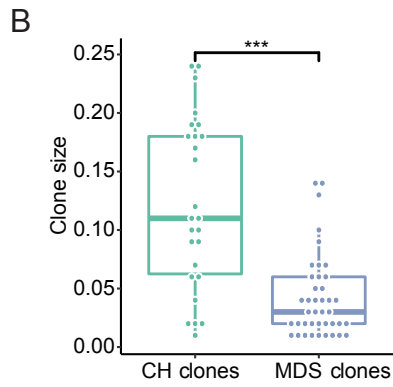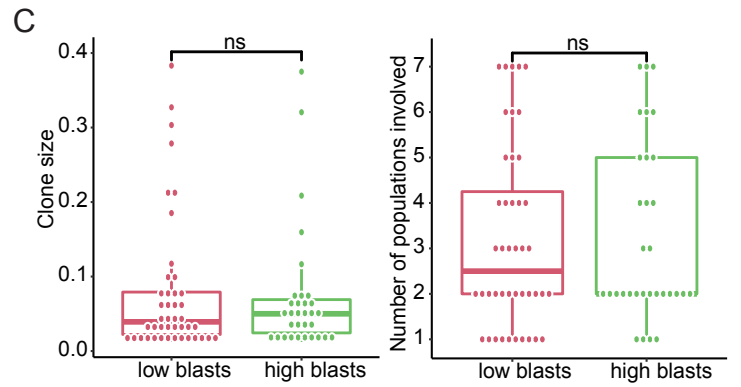

Supplement: Supplementary file 2 — Additional file 2: Figure S1. Gating strategy for seven cell populations. FACS strategy used to isolate single cells from the seven populations: H, CD34+CD45low cells; E, CD45−CD235a+CD71+ cells; M, CD45−CD235a−CD41+ cells; m, CD45+CD33+CD14+CD16− cells; n, CD45+CD33+CD14−CD16+ cells; B, CD45+CD19+CD20+ cells; and T, CD45+CD3+ cells. The upper panels represent HD data, and the lower panels represent data from a patient with MDS. Figure S2. Targeted single-cell RNA-seq method. A Schematic diagram of the targeted single-cell RNA-seq method. Single H, n, m, E, M, B, and T were sorted by flow cytometry into a 96-well plate. One-step RT-PCR was performed with the first-round primer mix. The second round of PCR was performed with PCR primer mix 1 or mix 2. Finally, a sequencing library was constructed with p5 and p7 adaptors. B Frequency at which the DNMT3A p.R301W mutation was detected in 32 single JURKAT cells. The positive rate was 78.1%. C Electrophoretogram of the representative libraries from single cells and 20 cells from a patient with MDS. One pair of each primer (U2AF1, SF3B1, ASXL1, TP53, TET2, and DNMT3A) from the PCR primer mix (PCR primer mix 1 or PCR primer mix 2) was used for the second round of PCR, followed by library construction. PCR products were separated on a gel. Library construction performed without cells served as a negative control (neg). Figure S3. Qualification of single cell RNA-seq data. A The results of single-cell filtration. The number of qualified and unqualified single cells is shown for individual donors. Only qualified cell data were used in the analysis. B Coverage of each mutation in single cells after filtration. The total number of reads covering each mutation site in qualified single cells is shown as the coverage. The threshold of coverage was 10x and is marked by a horizontal dotted line. Data are presented as the medians with interquartile ranges. Data points that fell outside of the upper and lower whiskers were considered o [file 40164_2022_280_MOESM2_ESM.pdf]
